# Timeliness in chronic kidney disease and albuminuria identification: a retrospective cohort study

**DOI:** 10.1186/s12875-015-0235-8

**Published:** 2015-02-13

**Authors:** Simon DS Fraser, Julie Parkes, David Culliford, Miriam Santer, Paul J Roderick

**Affiliations:** Academic Unit of Primary Care and Population Sciences, Faculty of Medicine, University of Southampton, South Academic Block, Southampton General Hospital, Tremona Road, Southampton, Hampshire SO16 6YD UK; Academic Unit of Primary Care and Population Sciences, Aldermoor Health Centre, Aldermoor Close, Southampton, SO16 5ST UK

**Keywords:** Chronic kidney disease, Albuminuria, Risk, General practice

## Abstract

**Background:**

Chronic kidney disease (CKD) is predominantly managed in primary care in the UK, but there is evidence of under-identification leading to lack of inclusion on practice chronic disease registers, which are necessary to ensure disease monitoring. Guidelines for CKD patients recommend urinary albumin to creatinine ratio (uACR) testing to identify albuminuria to stratify risk and guide management. This study aimed to describe the pattern and associations of timely CKD registration and uACR testing.

**Methods:**

A retrospective cohort of individuals with incident CKD 3–5 (two estimated glomerular filtration rates (eGFR) <60 ml/min/1.73 m^2^ ≥ three months apart) between 2007 and 2013 was identified from a linked database containing primary and secondary care data. Descriptive statistics and Cox proportional hazards models were used to identify associations with patient characteristics of timely CKD registration and uACR testing (within a year of first low eGFR).

**Results:**

12,988 people with CKD 3–5 were identified from 88 practices and followed for median 3.3 years. During this time period, 3235 (24.9%) were CKD-registered and 4638/12,988 (35.7%) had uACR testing (median time to CKD registration 307 days and to uACR test 379 days). 1829 (14.1%) were CKD-registered and 2229 (17.2%) had uACR testing within one year. Amongst people whose CKD was registered within a year, 676/1829 (37.0%) had uACR testing within a year (vs. 1553/11,159 (13.9%) of those not registered (p < 0.001)). Timely uACR testing varied by year, with a sharp rise in proportion in 2009 (when uACR policy changed). Timely CKD registration was independently associated with lower eGFR, being female, earlier year of joining the cohort, having diabetes, hypertension, or cardiovascular disease but not with age. Timely uACR testing was associated with timely CKD registration, younger age, having diabetes, higher baseline eGFR and later year of joining the cohort.

**Conclusions:**

Better systems are needed to support timely CKD identification, registration and uACR testing in primary care in order to facilitate risk stratification and appropriate clinical management.

**Electronic supplementary material:**

The online version of this article (doi:10.1186/s12875-015-0235-8) contains supplementary material, which is available to authorized users.

## Background

There is strong evidence from international meta-analyses of cohort studies that low estimated glomerular filtration rate (eGFR) and albuminuria are independent risk factors for poor clinical outcomes including all-cause and cardiovascular mortality, progression of chronic kidney disease (CKD) and acute kidney injury (AKI) [1-3]. These associations exist in men and women, vary with age, and occur in people with and without diabetes or hypertension [4-8]. CKD and AKI are important causes of hospitalisation and high healthcare costs. The total cost of CKD to the English NHS in 2009/10 has been estimated at £1.44 billion, over half of which was related to renal replacement therapy (dialysis and transplant) [9-11]. Guidelines recommend urinary albumin to creatinine ratio (uACR) testing to identify albuminuria in CKD (rather than protein to creatinine ratio or dipstick testing), with subsequent use of renin-angiotensin aldosterone system inhibitors (RAASi) in people with moderate to heavy proteinuria as a means of reducing risk of CKD progression and cardiovascular disease (CVD) [12-14].

**Figure 1:**
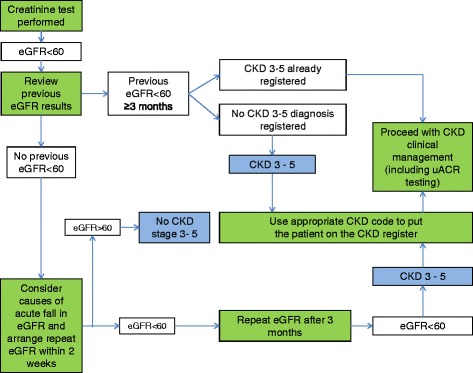
**Procedures leading to CKD registration.** Key: Green boxes indicate clinician actions, blue boxes indicate diagnostic points.

CKD identification has been the focus of several policy initiatives in the UK. The National Service Framework for Renal Services 2004/05 led to national reporting of eGFR by clinical biochemistry laboratories from 2006 [15], the General Practice pay for performance Quality and Outcomes Framework (QOF) included targets for specific CKD management indicators (following inclusion on a practice CKD register) from 2006/7 [16], the National Institute for Health and Care Excellence (NICE) introduced CKD guidelines in 2008 [12], and the NHS Vascular Checks Programme (from 2009) includes screening for CKD (stage 3–5) by eGFR measurement in people aged 35–74 with newly-identified type 2 diabetes or hypertension [17]. Despite these efforts, there is evidence that CKD is under-registered in Primary Care [18]. In 2013 NHS Kidney Care demonstrated wide variation in age-standardised prevalence of registered CKD between GP practices [19]. In England in 2012/13 the average registered CKD prevalence was 3.4% [20] compared with 7.3% in the Quality Improvement in CKD (QICKD) study (which used primary care biochemistry data to define CKD) [21], and 5.2% in the Health Survey for England [22]. In a recent large cohort study using routine data in the UK, about 28% of people with biochemically confirmed CKD were not labelled with a CKD Read diagnostic code (and therefore not registered) [23]. CKD diagnosis and registration require a logical series of steps to be taken by clinicians based on eGFR values (see Figure 1). Since Read codes assigned to the patient record by GPs lead to automatic registration, the timeliness of registration reflects the speed with which GPs recognize a new diagnosis of CKD. CKD under-registration may arise from diagnostic challenges, such as the need for repeat blood testing after three months, but clinicians’ concerns about CKD over-diagnosis, particularly in the elderly, and anxiety about communicating the diagnosis may also adversely influence CKD registration [24,25].

Since 2009, uACR testing has been included in Quality and Outcomes Framework CKD targets (although the diabetes domain has included microalbuminuria testing since 2004) [16], but target achievement data does not allow for assessment of the timeliness of CKD registration or uACR testing in relation to low eGFR results [20]. The aims of this study were to describe the associations of firstly CKD registration and secondly uACR testing within one year of first low eGFR in a population of people with biochemically-defined CKD.

## Methods

A retrospective cohort of people over 18 with incident CKD between 01/01/2007 and 16/05/2013 was identified from routine clinical biochemistry data using the Hampshire Health Record analytical database (HHR). The HHR is a shared clinical record containing anonymised individual linked extracts of clinical records from approximately 150 GP practices in Hampshire and two hospitals (University Hospital Southampton NHS Foundation Trust and Portsmouth Hospitals NHS Trust), including pathology results, with a total registered population of >1.1 million people. Changes in practice clinical computer systems can lead to variable ability to feed data to the HHR and practices without consistent pathology data throughout the study period were excluded.

Incident CKD stage 3 – 5 was defined as two values of eGFR < 60 ml/min/1.73 m^2^ at least three months apart (with no prior CKD diagnosis) according to guidelines [12-14]. People with intervening eGFR values > 60 ml/min/1.73 m^2^ were not considered as having CKD to exclude people with transient falls in eGFR. Study entry was defined as date of first eGFR < 60 ml/min/1.73 m^2^ because this indicated the earliest identifiable point of the incident CKD occurrence and represents the earliest point at which a clinician could have observed an abnormality of renal function. The clinical biochemistry departments confirmed that, during the study period, eGFR was calculated using the simplified Modification of Diet in Renal Disease equation and creatinine assays conducted using standardised Jaffe’s assay method, with calibration traceable to a standard reference material (isotope dilution-mass spectrometry) [26-28].

Age was defined as age at study entry. Socioeconomic status was assessed using quintiles of the England rank of the Index of Multiple Deprivation (IMD, 1 = most deprived, 5 = least deprived) as single entry at data extraction [29]. Ethnicity was poorly recorded in these data (estimated <40%) and was not included in analyses. Relevant clinical diagnoses were identified and classified using standard Read code hierarchy lists [30]. CKD registration status (registration / non-registration) was identified using CKD stage 3–5 Read codes, and uACR testing status from pathology records (see Additional file 1 for Read codes used). Hypertension, diabetes and CVD were defined by history of the diagnosis (presence of relevant Read code) in GP records at study entry. Diabetes included all type 1 and type 2 codes and CVD included codes for cerebral infarct, cerebral thrombosis, ischaemic heart disease, hypertensive heart disease, intracerebral haemorrhage, stroke, transient ischemic attack, or peripheral vascular disease. CVD was included because of the high degree of CVD comorbidity in this predominantly elderly population. It was also perceived that the presence of any of these comorbidities may influence CKD registration behaviour among clinicians. Mortality was defined by death recorded in the GP or hospital record.

### Ethics

Use of the Hampshire Health Record for research is regulated by the Hampshire Health Record Advisory Group (HHRAG) and the NHS South Commissioning Support Unit Business Intelligence team. HHR data is pseudonymized and therefore it is not possible to identify patients. These governance mechanisms mean that data from the HHR can be examined with HHRAG approval but without the need for formal ethical approval.

### Statistical analyses

Descriptive statistics were used to summarise the characteristics of the study population by their CKD registration status i.e. registered as having CKD for the Quality and Outcomes Framework or tested for uACR between 01/01/2007-16/05/2013 and the primary outcome of timely registration and uACR testing (defined as occurrence within a year of the date of first eGFR < 60 ml/min/1.73 m^2^). Chi squared test was used to compare categorical variables. Univariate, age-sex adjusted and multivariable Cox regression was used to identify the associations of timely CKD registration and uACR testing. Cox proportional hazards assumptions were checked. A time period of one year from study entry was chosen because we considered this a reasonable time to allow for repeat eGFR testing after three months (to confirm CKD) and a further nine months to allow for CKD registration and a uACR testing. Participants were censored at one year or at the end of registration with the last registered practice (for example if they moved away), death, or 16/05/2013 (data extraction date) if these occurred sooner. (Analyses conducted on those moving away within a year or on those joining the cohort at the end of 2012 may therefore not have allowed a full year of follow up).

To allow for the possibility that proteinuria had been assessed using urinary protein to creatinine ratio (uPCR) rather than uACR, we also identified those tested for uPCR between 01/01/2007-16/05/2013 and conducted sensitivity analyses of the Cox regression models with ‘uACR OR uPCR within a year’ as the outcome of interest.

Analyses were conducted using STATA version 12.1 and SAS version 9.3.

## Results

**Figure 2:**
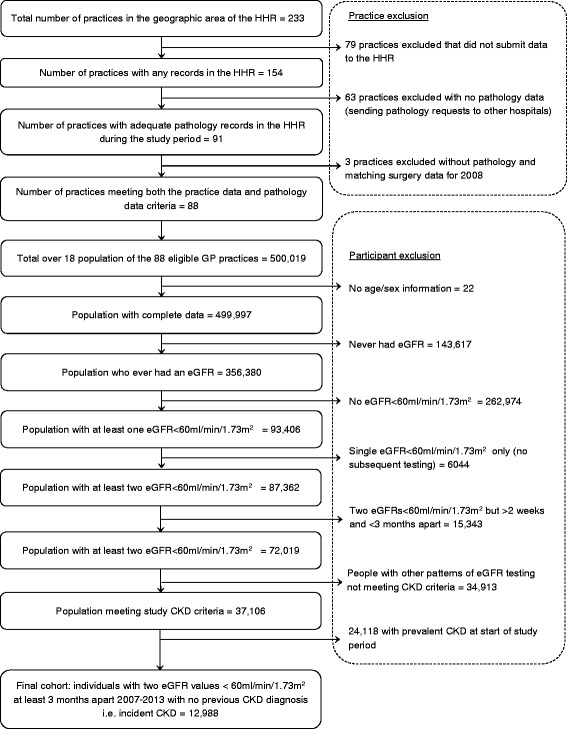
**Flow chart of study population identification in the Hampshire Health Record (HHR).**

Of the 499,997 people with complete data, 93,406 (18.7%) had at least one eGFR < 60 ml/min/1.73 m^2^. Only a small number of those (6044, 6.5%) had no further eGFR testing (Figure 2). We identified 12,988 people who met the criteria for incident CKD 3–5 (Figure 2) and followed them up for a median of 3.3 years.

### CKD registration within a year

Of the incident CKD study population, 3235/12,988 (24.9%) people were CKD-registered at some point between 2007 and 2013 (median time to registration 307 days (interquartile range (IQR) 100 to 629 days). Of those who were ever CKD-registered, 1829/3235 (56.5%) were registered within one year of their first low eGFR (Table 1). This represented 14.1% of the total study population with CKD (n = 12,988). A higher proportion of people with poor baseline renal function (eGFR <30 ml/min/1.73 m^2^ i.e. CKD 4–5) were CKD-registered within a year (52/327; 15.9%) than those with better renal function (eGFR ≥45 ml/min/1.73 m^2^ i.e. CKD 3a, 1542/11,450; 13.5%, p < 0.001). A higher proportion of men and people with diabetes, hypertension or CVD were CKD-registered within a year. CKD registration within a year did not vary significantly by deprivation status (Table 1). On univariate Cox regression analysis, women, people with comorbidities (diabetes, hypertension or CVD) and people with lower eGFR were more likely to be CKD-registered within one year. People study-diagnosed as CKD in later years of the cohort were less likely to have timely CKD-registration. These associations remained after adjustment for diabetes, hypertension, CVD, baseline eGFR, deprivation and year of joining the cohort (Table 2). Quality and Outcomes Framework ‘exception reporting’ for CKD was identified in 354 people (2.7%). ‘Exception reporting’ in the Quality and Outcomes Framework allows clinicians to exclude patients from recall (though they have CKD) in particular circumstances where this would be inappropriate, such as terminal illness or extreme frailty.

**Table 1:** **Characteristics of the study population (incident CKD) by CKD registration status**

**Variable**	**Category**	**CKD registered within 1 year (n = 1829)**		**Not CKD registered within 1 year (n = 11,159)**		**Total (n = 12,988)**		
		**n**	**Column %**	**n**	**Column %**	**n**	**Column %**	**p value**
**Sex**	Male	836	45.7	4627	41.5	5463	42.1	0.001
	Female	993	54.3	6532	58.5	7525	57.9	
**Age at study entry**	<50	34	1.85	394	3.5	428	3.3	0.001
	50-69	470	25.7	2921	26.1	3391	26.1	
	70-79	699	38.2	4274	38.3	4973	38.3	
	80+	626	34.2	3570	32.0	4196	32.3	
**IMD quintile**	1 (most deprived)	156	8.5	1034	9.3	1190	9.2	0.33
	2	266	14.5	1536	13.8	1802	13.9	
	3	412	22.5	2324	20.8	2736	21.1	
	4	424	23.1	2674	24.0	3098	23.9	
	5 (least deprived)	570	31.2	3582	32.1	4152	32.0	
**CVD** at study entry	Yes	547	29.9	2927	26.2	3474	26.7	0.001
	No	1282	70.1	8232	73.8	9514	73.3	
**Hypertension** at study entry	Yes	1146	62.6	6049	54.2	7195	55.4	<0.001
	No	683	37.3	5110	45.8	5793	44.6	
**Diabetes** at study entry	Yes	344	18.8	1707	15.3	2051	15.8	<0.001
	No	1485	81.2	9452	84.7	10,937	84.2	
**eGFR at study entry** (ml/min/1.73 m^2^)	≥45	1542	84.3	9908	88.8	11,450	88.2	<0.001
	30-44	235	12.8	976	8.7	1211	9.3	
	15-29	40	2.2	182	1.6	222	1.7	
	<15	12	0.7	93	0.8	105	0.8	
**uACR test within 1 year**	Yes	676	37.0	1553	13.9	2229	17.2	<0.001
	No	1153	63.0	9606	86.1	10,759	82.8	

Abbreviations in Table 2: CKD chronic kidney disease, IMD Index of multiple deprivation, BP blood pressure, eGFR estimated Glomerular Filtration Rate, CVD cardiovascular disease.

**Table 2:** **Cox regression analysis of CKD registration within 12 months (1829 events of total n = 12,988)**

**Variable**	**Category**	**Univariate**			Multivariable*		
		**HR**	**(95%** **CI)**	**p**	**HR**	**(95%** **CI)**	**p**
**Sex** (female vs. male)		1.17	(1.10-1.27)	0.001	1.19	(1.08-1.30)	0.001
**Age** (continuous)		1.00	(1.00-1.01)	0.058	1.00	(1.00-1.01)	0.546
**IMD quintile** (vs. least deprived)	1 (most deprived)	0.95	(0.79-1.13)	0.324	0.92	(0.88-1.13)	0.381
	2	1.08	(0.94-1.25)		1.07	(0.96-1.23)	
	3	1.10	(0.97-1.25)		1.09	(0.92-1.23)	
	4	1.00	(0.88-1.13)		0.99	(0.77-1.11)	
**Cardiovascular disease at baseline** (vs no CVD)		1.19	(1.10-1.29)	0.001	1.11	(1.00-1.23)	0.049
**Diabetes at baseline** (vs. no diabetes)		1.25	(1.10-1.32)	0.001	1.21	(1.08-1.37)	0.002
**Hypertension at baseline** (vs. no hypertension)		1.39	(1.14-1.32)	<0.001	1.36	(1.23-1.49)	<0.001
**Baseline eGFR** (continuous, ml/min/1.73 m^2^)		0.99	(0.99-1.00)	<0.001	0.99	(0.98-0.99)	<0.001
**Year of joining cohort** (vs. 2007)	2008	0.67	(0.53-0.84)	<0.001	0.71	(0.56-0.89)	<0.001
	2009	0.54	(0.43-0.69)		0.58	(0.46-0.74)	
	2010	0.42	(0.33-0.54)		0.44	(0.34-0.57)	
	2011	0.46	(0.36-0.60)		0.49	(0.38-0.63)	
	2012	0.41	(0.33-0.57)		0.46	(0.34-0.60)	

*adjusted for age, sex, index of multiple deprivation, cardiovascular disease, hypertension, diabetes, baseline eGFR, year of joining cohort.

Abbreviations in Table 3: CKD chronic kidney disease, IMD Index of multiple deprivation, BP blood pressure, eGFR estimated Glomerular Filtration Rate, CVD cardiovascular disease.

### uACR and uPCR testing within a year

In the study population, 4638/12,988 (35.7%) people had a uACR test between 2007 and 2013, and 2229/12,988 (17.2%) people were tested within a year. Median time to first uACR test was 379 days (IQR 150 to 715 days). By comparison, 965/12,988 (7.4%) people had a uPCR test between 2007 and 2013, and 429/12,988 (3.3%) people were tested within a year. Median time to first uPCR test was 413 days (IQR 140 to 778 days). Both uACR and uPCR had been tested in 529/12,988 (4.1%) people and 101 (0.8%) had both tested within a year. A total of 5074/12,988 (39.1%) had some form of laboratory proteinuria assessment (i.e. uACR or uPCR) of whom 2557/12,988 (19.7%) had it within a year.

**Figure 3:**
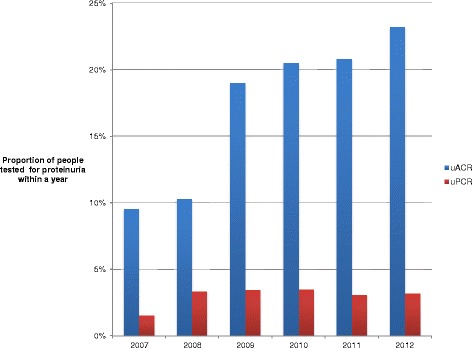
**Proportion of people with uACR and uPCR testing within a year by year of joining the cohort.**

Although the overall proportion of people tested within a year remained below 25%, there was an increase over time with a notable step change in 2009 (Figure 3). Among the 1829 people whose CKD was registered within a year, 676 (37.0%) also had uACR testing within a year (vs.1553/11,159 (13.9%) of those not registered within a year). On univariate analyses, females, younger people, people from less deprived areas, people with higher baseline eGFR, people with diabetes, people with CVD, people joining the cohort later and people whose CKD was registered within one year were more likely to have timely uACR testing (Table 3). These associations were maintained for younger people, diabetes, baseline eGFR, later entrants to the cohort, and people whose CKD was registered within one year in the fully adjusted model. The associations were similar in the sensitivity analyses (with uACR or uPCR within a year as the outcome of interest) except that female gender and hypertension were independently associated with timely proteinuria testing.

**Table 3:** **Cox regression analysis of uACR testing within 12 months (2229 events of total n = 12,988)**

**Variable**	**Category**	**Univariate**			Multivariable*		
		**HR**	**(95%** **CI)**	**p**	**HR**	**(95%** **CI)**	**p**
**Sex** (female vs. male)		1.42	(1.31-1.54)	<0.001	1.09	(1.00-1.19)	0.438
**Age** (continuous)		0.99	(0.98-0.99)	<0.001	0.99	(0.99-1.00)	0.028
**IMD quintile** (vs. least deprived)	1 (most deprived)	1.20	(1.03-1.40)	<0.001	0.88	(0.75-1.02)	0.135
	2	1.29	(1.13-1.46)		1.05	(0.92-1.19)	
	3	1.13	(1.01-1.27)		0.98	(0.87-1.10)	
	4	0.97	(0,86-1.09)		0.91	(0.81-1.02)	
**Cardiovascular disease at baseline** (vs. no CVD)		1.27	(1.16-1.39)	<0.001	0.948	(0.86-1.04)	0.260
**Diabetes at baseline**(vs. n diabetes)		10.24	(9.41-11.14)	<0.001	9.32	(8.54-10.18)	<0.001
**Hypertension at baseline** (vs. no hypertension)		1.46	(1.34-1.59)	<0.001	1.06	(0.97-1.16)	0.215
**Baseline eGFR** (continuous)		1.01	(1.01-1.02)	<0.001	1.01	(1.00-1.01)	0.014
**Year of joining cohort** (vs. 2007)	2008	1.08	(0.75-1.56)	<0.001	1.24	(0.86-1.79)	<0.001
		2009	2.09	(1.46-2.99)		2.56	(1.78-3.68)
		2010	2.30	(1.60-3.30)		2.65	(1.84-3.82)
		2011	2.34	(1.63-3.38)		2.73	(1.89-3.94)
		2012	2.65	(1.83-3.83)		2.54	(1.75-3.69)
**CKD registration within a year (vs. not)**		2.94	(2.69, 3.22)	<0.001	2.79	(2.55-3.06)	<0.001

*adjusted for age, sex, index of multiple deprivation, cardiovascular disease, hypertension, diabetes, baseline eGFR, year of joining cohort, CKD registration within 1 year.

Abbreviations in Table 3: CKD chronic kidney disease, IMD Index of multiple deprivation, BP blood pressure, eGFR estimated Glomerular Filtration Rate, CVD cardiovascular disease.

## Discussion

This study has shown in a large population of people with incident CKD defined from routine biochemistry data, timely CKD registration and uACR testing were poor. Only a quarter were registered as having CKD (CKD registration) after median follow up of about 3 years and 14.1% were registered within a year of first low eGFR (median time to CKD registration 0.8 years). Women, people with hypertension, diabetes or CVD and people with lower eGFR were more likely to have been CKD registered within a year. Age was not associated with CKD registration, but people with incident CKD from 2008 onwards were less likely to be registered within a year. The reasons for this are unclear and merit further investigation, particularly in the context of an increase in uACR testing following the change in incentives in 2009 (to include uACR, though there was no change in the CKD registration incentive). About a third of people with study-identified CKD had uACR tested at some point during the study period and 8% were tested within a year of first low eGFR (median time to uACR test 1.5 years). Younger age, diabetes, having timely CKD registration and joining the cohort later were positively associated with uACR testing. Associations with timely uACR *or* uPCR testing also included female sex and hypertension, although numbers tested for uPCR were low compared with uACR.

### Strengths and limitations

Strengths included the large study size, its population-based approach, and use of biochemically-defined CKD (with a conservative CKD definition). There were several limitations. Use of routine data may lead to underestimation of true CKD incidence (case identification relied on routine blood testing, though testing is higher in older age groups who have the highest prevalence) [31]. Missing data precluded assessment of the influence of ethnicity on CKD registration and uACR testing, although the study area has a lower than national ethnic minority population prevalence. This limits the generalisability of this study to areas with greater ethnic diversity. We could not identify changes in Index of Multiple Deprivation (linked to area of residence) over time, potentially misclassifying deprivation status in some who moved during the study. Other proteinuria testing methods (such as urine dipstick) were not assessed; we may therefore have underestimated efforts to ascertain proteinuria status. However, our research question related to uACR / uPCR testing as this is recommended in CKD guidelines [12,16]. Restricting inclusion of general practices to those with available data across the whole study period improved validity of results, but reduces generalisability. An important limitation is that we were unable to identify registered practice or individual GP at date of first low eGFR, so could not include these as variables in our models. We were therefore unable to adjust for differing GP or practice characteristics that may have influenced CKD registration or to account for clustering within practice. Finally, it was beyond the remit of this study to compare other quality of care measures, such as BP control, CVD risk assessment, statin and RAASi use in those registered/not registered. These are important considerations for future research.

### Comparison with existing literature

To our knowledge, this is the first study to explore factors associated with timely CKD registration and uACR testing. The New Opportunities for Early Renal Intervention by Computerised Assessment (NEOERICA) study identified a higher prevalence of biochemically-defined than diagnosed renal disease (8.5% vs. 1.6% respectively) [32] but NEOERICA was carried out prior to the introduction of CKD in the Quality and Outcomes Framework and is therefore not directly comparable. Following its inclusion in the Quality and Outcomes Framework, CKD registration increased in primary care (from 2.4 to 4.3% between 2007 and 2011) [33], but our study demonstrates that many patients with probable CKD are not entered onto a CKD register or uACR tested so remain more likely to miss out on treatments that are effective in preserving renal function. McIntyre et al. [18] have shown that many patients identified with CKD could benefit from more intensive treatment, for instance closer BP control, but qualitative research has highlighted tensions about how best to implement structured care for CKD in primary care [25]. In 2013, NHS Kidney Care made several recommendations for GP practices to help CKD identification including conducting audit to search for ‘missing’ CKD patients and ensuring awareness of CKD among clinical staff. [19] Our study suggests that having hypertension may influence CKD registration. This is reassuring as good BP control is probably the most important primary care intervention for people with CKD [34,35]. The lack of association between age and CKD registration suggests that older age does not adversely influence clinical decision to register. Lower baseline eGFR was appropriately associated with greater likelihood of CKD registration.

Achievement of individual QOF clinical indicators, including uACR testing, relies on accurately identifying and coding the underlying condition (to establish the prevalent population). Unsurprisingly, our study suggested that CKD registration influenced uACR testing. Timely uACR testing was better from 2009, consistent with the introduction of uACR as a CKD QOF indicator that year. Better testing among people with diabetes (though still suboptimal) may reflect greater clinician concern about diabetic nephropathy than CKD per se and/or diabetes albuminuria testing guidelines predating CKD ones [36]. Variation in QOF achievement can also arise from differences in exception reporting behaviour (the removal of patients judged by GPs to be inappropriate from calculations of quality achievement) but this was thought to be unlikely to explain our findings as the proportion with exception reporting was low. It is reassuring that we found no evidence of a social gradient in uACR testing as albuminuria prevalence was higher in lower socioeconomic groups in the Health Survey for England [22], and the QICKD study identified an association between lack of renal function monitoring (including proteinuria assessment) and adverse outcomes [37].

### Implications for research and practice

Delay or failure in CKD registration and uACR testing has implications for quality of care in people with CKD by reducing the possibility of early intervention to reduce future risk. In the context of growing pressures on primary care teams and the complexity of diagnosing CKD, our study supports the use of electronic record searching using tools such as IMPAKT™ to identify CKD rather than relying on manual coding [38]. This supports a similar recommendation made by Jain and colleagues in their recent UK-based cohort study, who identified that those not coded as having CKD received sub-optimal care [23]. Given the importance of albuminuria in risk stratification and as a prognostic indicator of CVD and AKI, the association between uACR testing and CKD registration in this study is a strong argument for improving timely CKD registration [1-8,39]. CKD identification with registration is more likely to lead to interventions such as assessment of cardiovascular risk [40,41], control of blood pressure [42], use of RAASi (based on albuminuria status) [43] and lipid lowering therapies in those with elevated CVD risk [44]. CKD registration and uACR measurement are therefore important quality issues that appropriately form part of the National CKD audit in England and Wales. Our study suggests that equity by gender should be an important consideration. In light of the high cost of renal replacement therapy, clarifying the extent to which the identification of CKD and albuminuria and their appropriate management has the potential to reduce CKD progression to end stage renal disease represent areas for future investigation. This study also highlights the need to investigate outcomes among people with different patterns of eGFR testing, such as those who only had one low eGFR with no subsequent investigation and those with transient eGFR changes, some of whom may have community-acquired acute kidney injury [45].

## Conclusions

This study has identified the need to strengthen systems to identify/register CKD and test for albuminuria in a timely fashion in primary care. Risk stratification is greatly improved by recording both eGFR and uACR status in people with CKD. Better systems of CKD identification and albuminuria assessment would enhance risk-reducing efforts.

### Availability of supporting data

These data are not publicly available in their current form, but access to anonymised data may be available by application to the authors and to the Hampshire Health Record Advisory Group.

## Additional file

Additional file 1:
**Read codes used.**

## Abbreviations

CKD: Chronic kidney disease
NHS: National Health Service
uACR: Urinary albumin to creatinine ratio
eGFR: Estimated glomerular filtration rate
AKI: Acute kidney injury
RAASi: Renin-angiotensin aldosterone system inhibitors
CVD: Cardiovascular disease
NICE: National Institute for Health and Care Excellence
HHR: Hampshire Health Record
IMD: Index of Multiple Deprivation
uPCR: Urinary protein to creatinine ratio
NEOERICA: New Opportunities for Early Renal Intervention by Computerised Assessment
GP: General practitioner
BP: Blood pressure
QICKD: Quality improvement in CKD
QOF: Quality and Outcomes Framework
